# Epitope mapping targeting the K205R protein of African swine fever virus using nanobody as a novel tool

**DOI:** 10.1128/spectrum.01750-24

**Published:** 2025-04-02

**Authors:** Xuedan Wei, Fengxia Zhang, Qiming Pei, Aijuan Shen, Duoxing Niu, Yaci Zhang, Ziheng Zhang, Yunshuo Lu, Angke Zhang, Gaiping Zhang, Hong Duan

**Affiliations:** 1 College of Veterinary Medicine, Henan Agricultural University, Zhengzhou, China; University of Prince Edward Island, Charlottestown, Prince Edward Island, Canada

**Keywords:** B-cell epitopes, K205R, African swine fever virus

## Abstract

African swine fever (ASF) is a highly infectious and lethal swine disease, leading to enormous losses in the pig industry. K205R, a non-structural protein of ASF virus (ASFV), is abundantly expressed at the early stages of viral infection and induces a strong immune response. In our previous study, five strains of K205R-specific nanobodies (Nbs) were screened through phage display technology, among which Nb1, Nb14, Nb35, and Nb82 exhibited good affinity. In the present study, the above four Nbs were successfully expressed in HEK293T cells and exhibited strong reactivity. Four Nbs recognized linear B-cell epitopes of K205R in both prokaryotic and eukaryotic expression systems. Besides, four Nbs specifically reacted with the K205R protein of ASFV-infected cells. Two epitopes ^1^MVEPR^5^ and ^188^RTQF^191^ were further identified, with highly conserved in different ASFV strains, and could interact with inactivated ASFV-positive sera, indicating that the two epitopes were natural linear B-cell epitopes. Moreover, structural analysis indicated that both epitopes were exposed on the surface of the K205R molecule. Notably, the identified epitope ^188^RTQF^191^ was first reported. Overall, these findings provide valuable insights for K205R as an effective diagnostic tool and vaccine development.

**IMPORTANCE:**

African swine fever (ASF) is the number one killer affecting the pig industry, and there are no effective strategies for prevention. The ASFV K205R protein is prominently expressed in the early stages of viral infection, triggering a robust immune response. The full understanding of K205R protein epitopes provides a theoretical basis for the development of vaccine-candidate proteins. Nanobodies exhibit superior capability in detecting concealed epitopes of antigens compared with traditional antibodies. Here, we identify two epitopes ^1^MVEPR^5^ and ^188^RTQF^191^ based on nanobodies as a tool. Notably, the epitope^188^RTQF^191^ is being reported for the first time. These epitopes are highly conserved in different ASFV strains and represent natural linear B-cell epitopes. This study opens up nanobodies as a new tool for the identification of epitopes and also provides a direct material basis for the development of ASFV vaccines.

## INTRODUCTION

African swine fever (ASF) is an acute, highly contagious, and fatal infectious disease caused by ASF virus (ASFV). ASF presents a significant threat to the global swine industry owing to its high morbidity and mortality rates of ≤100% (1). The disease is classified as a legally reportable animal disease by the World Organization for Animal Health, and it is also considered a class I animal disease that requires focused prevention measures in our country (2). Currently, no effective vaccine or drug is available against ASFV; therefore, strict biosafety measures are the most effective way to prevent and control ASF.

ASF was first reported in Kenya in East Africa in 1921 and has since become endemic in >30 countries and regions across Europe, Central America, South America, and beyond, posing a serious risk of further expansion (3, 4). In 2007, genotype II ASFV was first introduced into Georgia, outside of Africa, and then spread to other Caucasian and European countries (5). In August 2018, the first phase of ASF was reported in Shenyang (Liaoning, China), and the disease quickly spread to the whole country, causing a devastating blow to China’s pig industry (6, 7).

ASFV is the only known member of the family *Asfarviridae* and genus *Asfivirus* (5, 8). It is a giant and complex DNA virus of 170–193 kb in length containing >160 open reading frames (ORFs) encoding mainly 150–167 proteins (9). However, the functions of the majority of proteins remain unclear and require further exploration. Among these proteins, K205R is a non-structural protein encoded by ORF K205R of ~33 kDa in size with good immunogenicity (10). K205R, an early expression viral protein containing conserved amino acid sequences, begins 4 h following infection of cells by the virus (11). Notably, the K205R protein elicits a strong immune response in pigs, serving as a key antigen of ASFV (10, 12). Moreover, it has been identified as a potential early serodiagnostic antigen capable of detecting IgM response as early as 11 days post-infection with ASFV (13). However, the specific role of K205R in immune protection against ASFV remains unclear, and the identification of its dominant conserved epitopes establishes a crucial groundwork for serological diagnosis and vaccine development efforts for ASF.

Nanobodies (Nbs) are naturally occurring specific antibodies, derived from the heavy chain antibody variable region (VHH) in camelids, which lack the light chain and CH1 regions of the heavy chain (14). Nbs are screened from a VHH phage library using phage display technology after 3–4 rounds of panning, and the screening process is relatively brief. Nbs can be produced in various expression systems, including prokaryotic, eukaryotic, and yeast expression systems (15). Due to their small size of only 15 kDa, Nbs are easily amenable to genetic modifications and can be conjugated with ferritin and horseradish peroxidase (HRP) (16, 17). Nbs consist of three complementary determination regions (CDRs) and four framework regions (FRs), exhibiting ≤80% homology in their FRs, with CDR3 being a highly variable region (18). VHH has long CDR1 and CDR3 regions, with an average CDR3 length of 16 amino acids. The antigen-binding region of VHH tends to form a convex ring structure, providing Nbs with a distinct structural advantage over conventional antibodies for targeting clefts on antigenic surfaces (19).

In our previous study, an Alashan Bactrian camel was immunized with the ASFV K205R protein, and peripheral blood mononuclear cells were isolated from a blood sample after the last immunization to construct a VHH library. Five K205R-specific Nbs were screened using phage display technology. Among them, K205R-Nb1, -Nb14, -Nb35, and -Nb82 showed good binding activity and were used for subsequent experiments. In the present study, using the above four Nbs as tools, K205R epitopes were explored through truncated expression and synthetic polypeptides. Of note, Nb1, Nb14, and Nb82 recognized the same linear B-cell epitope, ^1^MVEPR^5^, while Nb35 recognized a B-cell epitope, ^188^RTQF^191^. The two epitopes were able to react with ASFV-positive sera from naturally infected pigs. The present findings will help to further understand the function of the K205R protein and provide a theoretical basis for the development of effective anti-ASFV drugs or diagnosis reagents using Nbs.

## MATERIALS AND METHODS

### Cells and sera

Human embryonic kidney 293T (HEK293T) and Vero cells were stored in the International Joint Research Center National Animal Immunology and propagated in Gibco DMEM (Invitrogen; Thermo Fisher Scientific, Inc.). Porcine alveolar macrophages (PAMs) were cultured in RPMI-1640 medium supplemented with 10% fetal bovine serum (HyClone; Cytiva) at 37˚C under a humid 5% CO_2_ atmosphere. Five ASFV antibody-positive sera were stored in our laboratory (20). Five ASFV antibody-negative sera were collected from ASF decontaminated pig farms and confirmed ASFV antigen and antibody-negative using reverse transcription-quantitative PCR and commercial ELISA kit detection (Beijing JinnuoBaitai Biotechnology Co., Ltd.).

### Production of Nbs-HRP fusion proteins against ASFV K205R protein

To identify linear B-cell epitopes on the ASFV K205R protein using Nbs, Nbs-HRP fusion proteins were generated. In a previous study, specific Nbs targeting the K205R protein were isolated through phage display technology and were designated as K205R-Nb1, K205R-Nb14, K205R-Nb35, K205R-Nb64, and K205R-Nb82. Out of these, K205R-Nb1, K205R-Nb14, K205R-Nb35, and K205R-Nb82 exhibited strong binding capabilities and were selected for further experiments. Subsequently, a secretion signal sequence (human Igκ), the Nbs, and HRP were inserted into the pCAGGS-HA vector using *EcoRI* and *NheI* restriction enzymes (New England BioLabs, Inc.) (21). The HEK293T cells were then cultured in six-well plates at a density of 2 × 10^5^ cells per well for 24 hours (h). Next, cells were transfected with pCAGGS-Nb1-HRP, pCAGGS-Nb14-HRP, pCAGGS-Nb35-HRP, and pCAGGS-Nb82-HRP recombinant plasmids to produce Nb-HRP fusion proteins using X-tremeGene HP DNA transfection reagent (Roche Diagnostics). The cell supernatant containing Nb-HRP fusion proteins was harvested after 60 h of transfection. Finally, the expression and secretion of ASFV-K205R-Nbs-HRP were evaluated using ELISA, western blotting, and indirect immunofluorescence assay (IFA).

### Affinity of Nbs-His binding to ASFV-K205R protein

ELISA was performed to evaluate the binding ability of Nbs-His protein to the K205R protein as previously described (22). Briefly, a 96-well ELISA plate was coated with K205R protein (400 ng/well) and incubated overnight at 4°C. After blocking and washing, Nb1-His, Nb14-His, Nb35-His, and Nb82-His proteins at various concentrations (ranging from 10 to 3 to 106 ng/well) were added to the respective wells and incubated for 1 h at room temperature (RT). Next, a mouse anti-camel polyclonal antibody (1:1,000) was incubated for 1 h at RT as the secondary antibody. Next, HRP-conjugated goat anti-mouse IgG (1:5,000; Jackson ImmunoResearch Laboratories, Inc.) was incubated for 1 h at RT as the third antibody. Finally, tetramethylbenzidine (TMB) was added to the plates (100 µL/well) for a colorimetric reaction, which facilitated the visualization and quantification of the binding interactions between Nbs-His and the K205R protein.

### Immunoperoxidase monolayer assay

Vero cells were cultured in 24-well plates at a density of 5 × 10^4^ cells per well for 24 h, and they were inoculated with the ASFV HLJ/18 strain at 0.01 multiplicity of infection (MOI). The samples were inactivated at 12 h post-infection. After blocking with 1% bovine serum albumin (BSA) at RT for 2 h, Nb1-, Nb14-, Nb35-, and Nb82-HRP fusions were added to the plates as the primary antibodies (diluted 1:100, 1:1,000, and 1:2,000, respectively) and incubated at 37°C for 30 min. Inactivated ASFV antibody-positive serum and ASFV antibody-negative serum served as controls. Subsequently, the samples were incubated with or without HRP-conjugated goat anti-swine IgG (1:1,000; Abcam) at 37 °C for 30 min. Next, 30 µL 3-amino-9-ethylcazole (AEC) dye was added to each well and incubated at RT for 10 min. Next, the residual AEC was washed away, double-distilled water was added, and the samples were observed and photographed using an inverted fluorescence microscope (Olympus Corporation). Cell samples of positive wells were brown-red, while cells of blank control and negative wells had no color when observed under the microscope.

All operations comply with biosafety standards. The cell samples were inactivated and were kindly provided by Harbin Veterinary Research Institute, which has a biosafety level 4 laboratory and can cultivate live ASFV, the samples were stored at −20°C during shipping.

### Eukaryotic expression and identification of recombinant ASFV K205R protein

To investigate the binding activity between Nbs-HRP and K205R, a eukaryotic expression system was established to express the K205R protein. The ASFV K205R gene, encoding the ASFV K205R protein, was amplified from the pET-30a-K205R template using E-K205R-F and E-K205R-R primer pairs (Table 1). Subsequently, the PCR products were purified and inserted into the pCAGGS-HA eukaryotic expression vector *via EcoR*I and *Xho*I restriction enzymes. Following verification through sequencing, the resulting plasmid was designated as pCAGGS-HA-K205R. The HEK293T cells were transfected with this recombinant plasmid using X-tremeGene HP DNA transfection reagent (Roche Diagnostics) to enable the expression of the eukaryotic K205R protein. At 48 h post-transfection, cells were either harvested or fixed for evaluation of K205R protein expression using western blotting and IFA.

**TABLE 1:** Primers used in this study^*a*^

Primer	Sequence(5′−3′)	Usage
Nbx-F	GGAATTCCATATGCAGGTGCAGCTGCAGG	pET21b-Nbs-His
Nbx-R	CCGCTCGAGTGAGGAGACGGTG	
K205R(1-138)-F	CGGGATCCATGGTTGAGCCACGCGAACAGT	pET30a-different truncated K205R
K205R(1-138)-R	CCGCTCGAGATTCGTGGGATTTTTTTTAGGTG	
K205R(69-206)-F	CGGGATCCATGCTTATGACCGTCATGACGGA	
K205R(69-206)-R	CCGCTCGAGCTTCTTCATCATCTCTTTGACCA	
K205R(34-138)-F	CGGGATCCATGAAAACGTCTTTTATGGTATCA	
K205R(34-138)-R	CCGCTCGAGATTCGTGGGATTTTTTTTAGGTG	
E-K205R-F	CCGGAATTC ATGGTTGAGCCACGCGAACAG	pCAGGS-HA- different truncated K205R
E-K205R-R	CCGCTCGAGTTACTTCTTCATCATCTCTT	
E-K205R(1-168)-F	CCGGAATTCATGGTTGAGCCACGCGAACAG	
E-K205R(1-168)-R	CCGCTCGAGTTACTCATCCAATATGGCTTG	
E-K205R(1-178)-R	CCGCTCGAGTTATCTTTCGATCCCGGTCTTG	
E-K205R(1-188)-R	CCGCTCGAGTTATCTCCACATGTAAAGACC	
E-K205R(1-198)-R	CCGCTCGAGTTACATTTTCTTCTGTTCGTCA	

^*a*^ Note: Restriction sites are underlined.

### Expression and purification of different truncated ASFV K205R and Nbs-His proteins

To identify epitopes recognized by the Nbs, various truncated K205R proteins were designed and expressed either using *E. coli* or an eukaryotic expression system. The genes encoding truncated K205R proteins (amino acids 1–138, 69–206, and 34–138) or (amino acids 1–168, 1–178, 1–188, and 1–198) were amplified using pET-30a-K205R as the template. Subsequently, the PCR products corresponding to amino acids 1–138, 69–206, and 34–138 were purified and cloned into the pET-30a vector (Novagen). After sequencing, the positive plasmids were transformed into *E. coli* BL21 (DE3) (TransGen Biotech, Co., Ltd.). Next, these proteins were expressed and purified following established methods (21). The remaining PCR products encoding amino acids 1–168, 1–178, 1–188, and 1–198 were also purified and cloned into the pCAGGS-HA eukaryotic expression vector. After sequencing, the positive plasmids were transfected into HEK293T cells to produce truncated K205R proteins *via* eukaryotic expression. The primer sequences for PCR amplification are listed in Table 1. Complete and truncated K205R proteins were analyzed using SDS-PAGE and used as antigens for western blotting, ELISA or IFA to determine their interaction with the Nbs and to identify the recognized epitope.

To further identify the affinity of the Nbs to the K205R protein, four Nbs were expressed and purified using a prokaryotic system. Nb1, Nb14, Nb35, and Nb82 were amplified using pCAGGS-Nb1-, -Nb14-, -Nb35-, and -Nb82-HRP as the template, respectively. Next, the PCR products were purified and cloned into the pET-21b prokaryotic expression vector using *Nde*I and *Xho*I restriction enzymes. After being sequenced, the positive plasmids were transformed into *E. coli* BL21 (DE3) (TransGen Biotech, Co., Ltd.). Next, these proteins were expressed and purified as previously described (23).

### Western blotting

HEK293T cells were harvested and lysed using RIPA buffer (Beyotime Institute of Biotechnology). Total cellular proteins (20 µg) were separated by 12.5% SDS-PAGE and transferred to polyvinylidene fluoride (PVDF) membranes (Millipore, Sigma). The PVDF membranes were then blocked with 5% skimmed milk in phosphate-buffered saline (PBS) with 0.01% Tween-20 (PBST) and incubated overnight at 4°C. Subsequently, the PVDF membranes were incubated with Nbs-HRP fusion proteins (1:1,000) for 1 h at 37°C. Following three washes with PBST, the membranes were visualized using an ECL substrate (Beijing Solarbio Science & Technology Co., Ltd.).

In a separate step, the PVDF membranes were blocked and subsequently incubated with primary antibodies for 2 h at RT, specifically, mouse anti-HA monoclonal antibody (mAb; 1:2,000; Proteintech Group, Inc.) and anti-α-tubulin (1:5,000; Proteintech Group, Inc.). After washing three times with PBST, HRP-conjugated goat anti-mouse IgG (1:5,000; Jackson ImmunoResearch Laboratories, Inc.) was incubated for 1 h at 37°C as the secondary antibody. Finally, the membranes were visualized using an ECL substrate (Beijing Solarbio Science & Technology Co., Ltd.). Chemiluminescence signal acquisition was performed using a ChemiDoc MP imaging system (Bio-Rad Laboratories, Inc.).

### IFA

The HEK293T cells were cultured in 24-well plates at a density of 5 × 10^4^ cells per well for 18 h. Subsequently, cells were transfected with pCAGGS-Nb1-, -Nb14-, -Nb35-, and -Nb82-HRP, or pCAGGS-HA-K205R and truncated K205R proteins (K205R-T4, -T5, -T6, and -T7) using X-tremeGene HP DNA transfection reagent (Roche Diagnostics). At 36 h post-transfection, the HEK293T cells were fixed with 70% ice-cold ethanol for 1 h at 4 ˚C, followed by blocking with 1% BSA for 1 h at RT. Next, cells were incubated with Nb-HRP fusions (1:500) as the primary antibody, washed three times with PBS, and further incubated with mouse anti-HA antibody for 1 h at 37°C (1:2,000, Proteintech Group, Inc.). Subsequently, the samples were incubated with a third antibody (Alexa Fluor 594-conjugated goat anti-mouse IgG [H&L]; 1:500; Abcam) for 1 h at 37°C. Finally, the nucleus was stained using DAPI (Thermo Fisher Scientific, Inc.) at RT for 5 min. Image acquisition was performed using a fluorescence microscope (Olympus Corporation).

PAMs (1 × 10^6^ cells/mL) were plated in a 24-well plate for 4 h, and infected with ASFV HLJ/18 of 0.01 MOI for 12 h. Subsequently, cells were fixed using 4% paraformaldehyde for 30 min at RT, permeabilized using 0.3% Triton X-100 for 5 min at RT, and then blocked with 1% BSA for 1 h at RT. Next, cells were incubated with Nb1-, Nb14-, Nb35-, and Nb82-HRP fusions (1:500) for 1 h at 37°C as the primary antibody. Subsequently, the cells were incubated with mouse anti-HA antibody for 1 h at 37 ˚C (1:2,000; Proteintech Group, Inc.) as the secondary antibody, and finally, with a third antibody (Alexa Fluor 594-conjugated goat anti-mouse IgG [H&L]; 1:500; Abcam) for 1 h at 37˚C. The ASFV-infected cell samples were inactivated and kindly provided by Harbin Veterinary Research Institute.

### ELISA

For direct ELISA analysis of Nb-HRP fusions expression, HEK293T cells were transfected with the pCAGGS-Nb1-, -Nb14-, -Nb35-, and -Nb82-HRP recombinant plasmids to produce Nbs-HRP fusion proteins. After transfection for 60 h, the cell supernatant containing Nbs-HRP fusion proteins was collected for ELISA. Briefly, cell supernatants of different volumes (100 or 200 µL/well) were coated onto 96-well ELISA plates for 2 h at RT. After being blocked with 200 µL 2.5% (wt/vol) non-fat dry milk, the plates were washed three times with PBST and 100 µL/well TMB was added. Finally, 3 M H_2_SO_4_ (50 µL/well) was added to stop the colorimetric reaction, and the optical density (OD) at 450 nm values was read using an automated ELISA plate reader (BioTek; Agilent Technologies, Inc.).

For indirect ELISA (iELISA) of the reaction between K205R-peptides and ASFV-positive sera, 96-well ELISA plates were coated with K205R-peptides (4 µg/well) and incubated overnight at 4°C. After being washed three times with 0.5% PBST, the plates were blocked with 200 µL 2.5% (wt/vol) non-fat dry milk, and incubated with ASFV-positive sera (1:10) for 1 h at 37°C, followed by incubation with HRP-conjugated goat anti-swine antibody (1:5,000). Finally, TMB was added for colorimetric reaction development.

### Dot blot and peptide-based ELISA

In the dot blot assay, each peptide (2 µg) was spotted onto nitrocellulose membranes (cat. no. AE99; Schleicher & Schuell, Inc.). After drying, the membranes were blocked with 5% skimmed milk for 2 h at RT. Subsequently, the membranes were incubated with primary antibodies for 1 h at RT. Finally, the membranes were visualized using an ECL substrate (Beijing Solarbio Science & Technology Co., Ltd.).

In the peptide-based ELISA, 96-well ELISA plates were coated with K205R-peptides (4 µg/well) and incubated overnight at 4°C. The plates were incubated with Nb-HRP fusions (1:500; 100 µL/well) for 1 h at RT. Next, 100 µL/well TMB (Beijing Solarbio Science & Technology Co., Ltd.) was added to the plates, followed by the addition of 3 M H_2_SO_4_ (50 µL/well) to stop the colorimetric reaction. Next, the OD_450 nm_ values were read using an automated ELISA plate reader (BioTek; Agilent Technologies, Inc.) All peptides were synthesized by GenScript, and the purity of the synthetic peptides was ≥95%.

### Molecular docking of K205R peptide and Nb

To analyze the complex formed by the combination of the K205R peptide and Nb, molecular docking was conducted. Initially, the three-dimensional (3D) structures of the four Nbs were generated through homology modeling using the SWISS-MODEL online server. Subsequently, all epitopes were protonated at pH 7.4, and their structures were expanded to 3D using Open Babel (24). AutoDock Tools (ADT3) were applied to prepare and parametrize the receptor protein and ligands. Next, docking grid documents were created by AutoGrid of a sitemap, and a docking simulation was carried out using AutoDock Vina (1.2.0) (25, 26). Finally, the resulting optimal pose was selected for interaction analysis, and the protein-ligand interaction figure was created using PyMOL. The K205R epitope was shown as a cyan stick, while the Nb was represented as a slate cartoon model, and their binding sites were shown as magenta stick structures. The hydrogen bonds were depicted as yellow.

### Statistical analysis

All the above experiments were performed independently at least three times, to ensure that the results were reproducible. Statistical significance was determined by Student’s *t*-test when two groups were compared, or by one-way analysis of variance (ANOVA) when more than two groups were compared. A *P*-value of <0.05 was considered to be statistically significant.

## RESULTS

### Production of Nbs-HRP fusion proteins against the ASFV K205R protein

**Fig 1:**
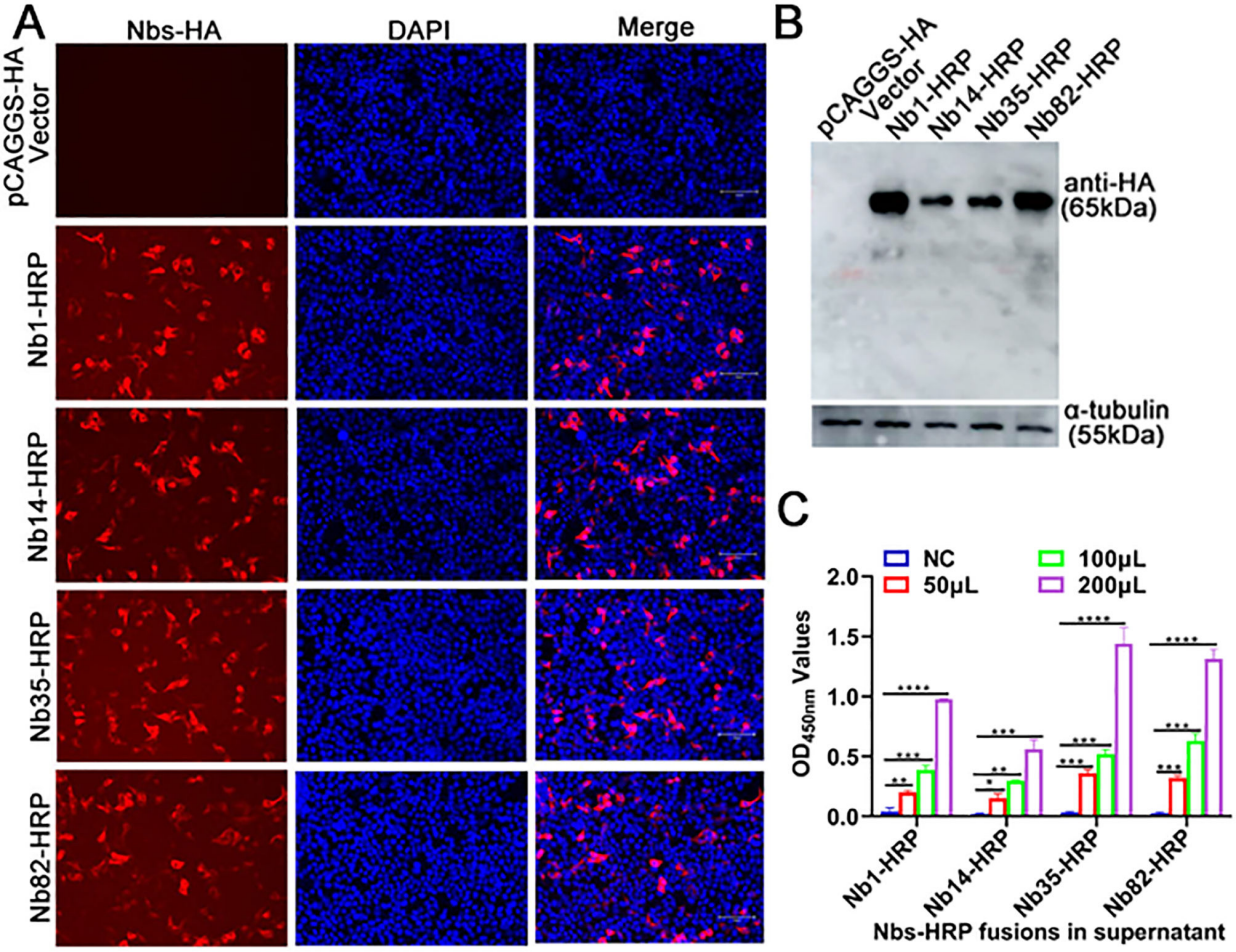
Production and identification of Nb-HRP fusion proteins, expressed and secreted, against ASFV K205R protein. The pCAGGS-HA-Nb1-, -Nb14-, -Nb35-, and -Nb82-HRP recombinant plasmids were transfected into HEK293T cells for 60 h to produce Nb-HRP fusion proteins. The HEK293T cells were fixed or collected 60 h after transfection, and the expression of Nb-HRP fusions was analyzed using (**A**) immunofluorescence assay and (**B**) western blotting with anti-HA monoclonal antibody as the primary antibody. (**C**) Cell culture supernatants were collected 60 h after transfection or not, and detection of HRP activity in the Nb-HRP fusions secreted into the cell culture supernatants was conducted using direct ELISA. Data are mean ± SD values of three independent results. ^*^P < 0.05; ^**^P < 0.01; ^***^P < 0.001;^****^P < 0.0001. Nb, nanobody; HRP, horseradish peroxidase.

To identify linear B-cell epitopes on the ASFV K205R protein by Nbs, Nb-HRP fusions were produced. In a previous study, five specific Nbs against the K205R protein were screened using phage display technology, and named K205R-Nb1, -Nb14, -Nb35, -Nb64, and -Nb82, respectively. Among them, K205R-Nb1, -Nb14, -Nb35, and -Nb82 exhibited strong binding activity and were selected for subsequent experiments (21). In the present study, HEK293T cells were transfected with pCAGGS-Nb1-, -Nb14-, -Nb35-, and -Nb82-HRP recombinant plasmids to produce Nbs-HRP fusion proteins. The IFA results confirmed the successful expression of Nb1-, Nb14-, Nb35-, and Nb82-HRP fusion proteins using anti-HA mAb as the primary antibody (Fig. 1A). Western blot analysis demonstrated that all four Nb-HRP fusions were successfully expressed in HEK293T cells as expected with a size of 65 kDa (Fig. 1B). Subsequently, the direct ELISA results indicated that the four Nb-HRP fusions could be detected by directly adding TMB, suggesting that the Nb-HRP fusions were successfully expressed and secreted in a dose-dependent manner (Fig. 1C).

### Reactivity of Nb1, Nb14, Nb35, and Nb82 with ASFV-K205R protein

**Fig 2:**
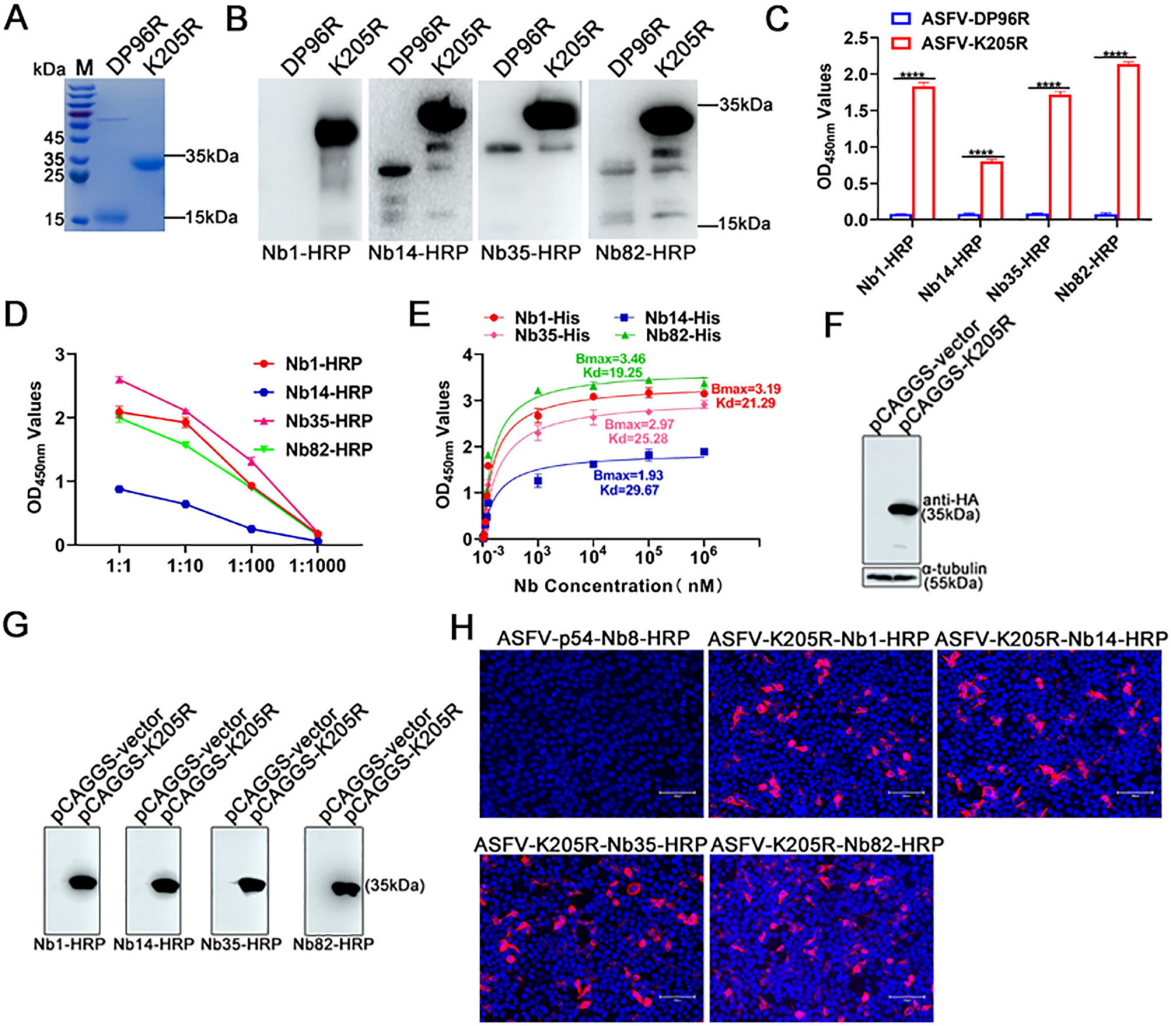
Four Nbs, namely Nb1, Nb14, Nb35, and Nb82, bind to both prokaryotically and eukaryotically expressed K205R proteins. (**A**) SDS-PAGE analysis of prokaryotically expressed ASFV-K205R protein. (**B**) Nb1, Nb14, Nb35, and Nb82’s reaction with ASFV-K205R protein expressed with *E. coli* was determined using western blotting, with ASFV-DP96R used as a negative control. (**C**) Four Nbs specifically reacted with the ASFV-K205R protein, as determined by direct ELISA. Data are mean ± SD values of three independent results. ^****^P < 0.0001. (**D**) Titration of cell supernatant containing Nb1-, Nb14-, Nb35-, and Nb82-HRP fusion proteins binding to the ASFV-K205R protein. (**E**) Affinity of Nb1-, Nb14-, Nb35-, and Nb82-His protein toward ASFV-K205R protein. (**F**) Western blot analysis of eukaryotically expressed ASFV-K205R protein using anti-HA monoclonal antibody for detection. Four Nbs specifically reacted with the eukaryotically expressed ASFV-K205R protein, as determined using (**G**) western blotting and (**H**) immunofluorescence assay. Nb, nanobody; ASFV, African swine fever virus.

The reactivity of Nb1, Nb14, Nb35, and Nb82 with ASFV-K205R protein was investigated through various experiments. First, SDS-PAGE analysis confirmed that the recombinant ASFV-K205R protein was successfully expressed with the expected size of 35 kDa, and the high purity of the target protein was obtained *via* Ni-resin purification (Fig. 2A). Subsequently, using this protein as the antigen, western blot assays demonstrated that Nb1, Nb14, Nb35, and Nb82 recognized the linear epitope of the K205R protein while showing no reactivity toward the ASFV-DP96R protein produced under the same system (Fig. 2B). Moreover, direct ELISA analysis revealed specific binding of Nb1, Nb14, Nb35, and Nb82 to the K205R protein, excluding ASFV-DP96R (Fig. 2C).

Further analysis of the Nbs’ affinity was conducted by ELISA with different dilution ratios of Nbs-HRP on the coated K205R antigen. Notably, the OD_450 nm_ values exceeded 1.0 at a 1:100 dilution of cell supernatants containing Nb1-, Nb35-, and Nb82-HRP fusion proteins, with Nb14-HRP demonstrated a relatively lower affinity compared to the other Nbs (Fig. 2D). These four Nbs were also expressed and purified using a prokaryotic system, followed by determination of their binding affinity, as shown in Fig. 2E. The affinity constants toward the K205R protein were found to be as follows: Nb1-His, 21.29 HAU mL; Nb14-His, 29.67 HAU mL; Nb35-His, 25.28 HAU mL; and Nb82-His, 19.25 HAU mL. To further confirm the binding capability of the Nbs to the K205R protein, a pCAGGS-HA-K205R eukaryotic expression vector was constructed and transfected into HEK293T cells. Western blot analysis demonstrated successful expression of the K205R protein with a clear band at ~35 kDa (Fig. 2F). Notably, all four Nbs exhibited binding to the eukaryotic expression K205R protein, as evidenced by western blotting (Fig. 2G) and IFA (Fig. 2H), thereby confirming their ability to target the specific antigen.

### Reactivity of Nbs-HRP with ASFV-infected cells

**Fig 3:**
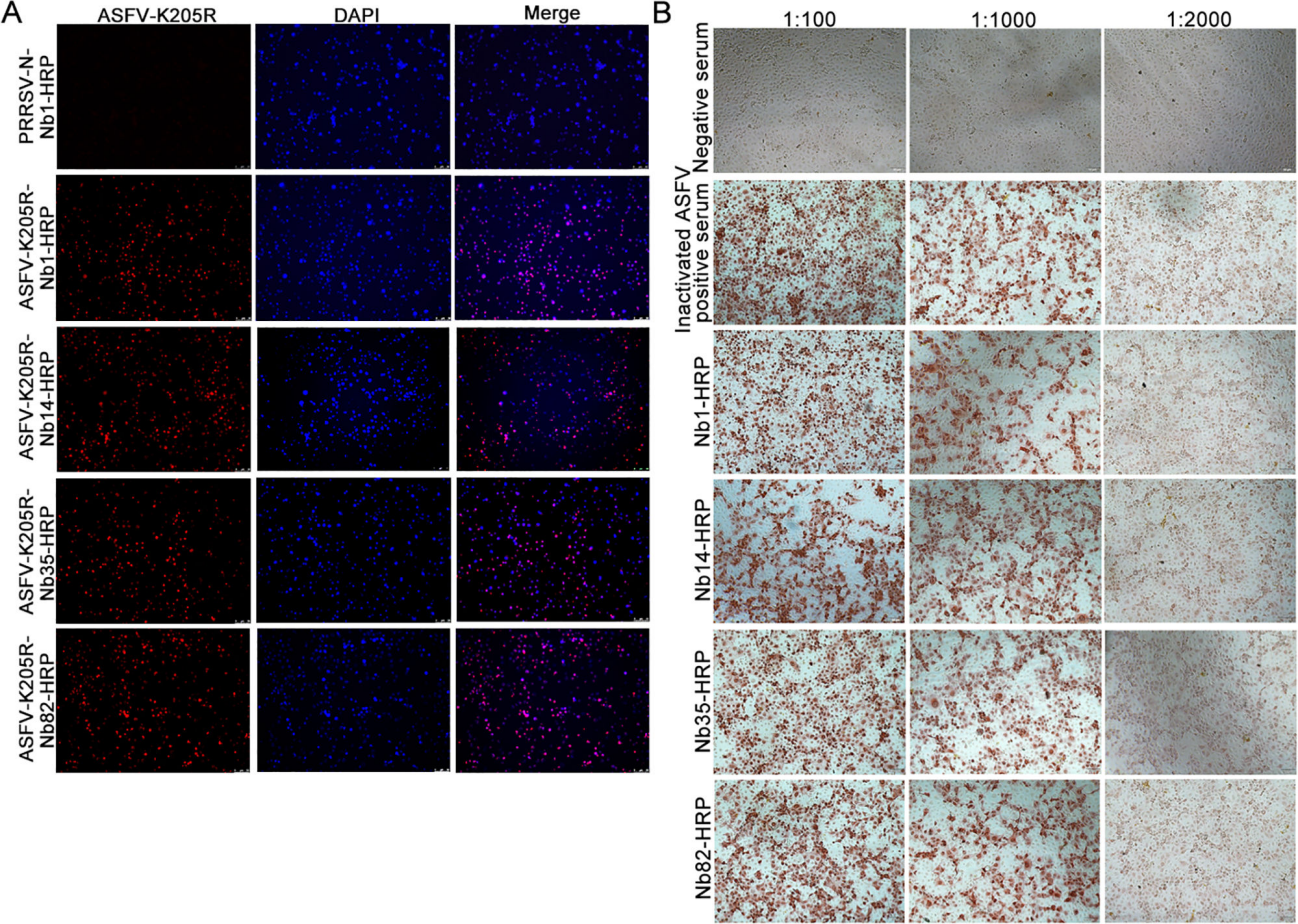
Nb1, Nb14, Nb35, and Nb82 can recognize ASFV strains HLJ/18-infected PAMs or Vero cells, according to by IFA and immunoperoxidase monolayer assay. (**A**) IFA analysis of the reaction of four Nbs with ASFV K205R. PAMs were plated and infected with the HLJ/18 ASFV strain at an MOI of 0.01 for 12 h. Next, PAMs were fixed and used for immunostaining analysis with Nbs-HRP as the primary antibody. (**B**) Immunoperoxidase monolayer assay identification of the reaction of the four Nbs with ASFV K205R. Vero cells were infected with the HLJ/18 ASFV strain at an MOI of 0.01 for 16 h. Next, the cells were fixed and incubated with Nbs-HRP supernatants at a serial dilution of 1:100, 1:1,000, and 1:2,000. Inactivated anti-ASFV antibody-positive serum was used as the positive control, while anti-ASFV antibody-negative serum was used as the negative control. Nb, nanobody; ASFV, African swine fever virus; IFA, immunofluorescence assay; MOI, multiplicity of infection.

To further verify that the Nbs could recognize ASFV K205R, ASFV infection assays were performed using IFA detection. As shown in Fig. 3A, Nb1-, Nb14-, Nb35-, and Nb82-HRP recognized ASFV-infected PAMs, while the control supernatant from PRRSV-N-Nb1-HRP did not react with ASFV-infected PAMs. These results confirmed that all four Nbs could recognize ASFV strains HLJ/18-infected PAMs.

Immunoperoxidase monolayer assay (IPMA) was also performed to determine the reactivity of the four Nbs toward ASFV HLJ/18 strains-infected Vero cells. As shown in Fig. 3B, Nb1-, Nb14-, Nb35-, and Nb82-HRP were able to react with ASFV-infected Vero cells. The IPMA titers of Nb1-, Nb14-, Nb35-, and Nb82-HRP were 1:1,000 (Fig. 3B), while the inactivated positive serum of recovered pigs was also 1:1,000 (Fig. 3B) but was unable to react with the virus.

### Identification of the epitope recognized by ASFV-K205R-Nb1, Nb14, Nb35, and Nb82

**Fig 4:**
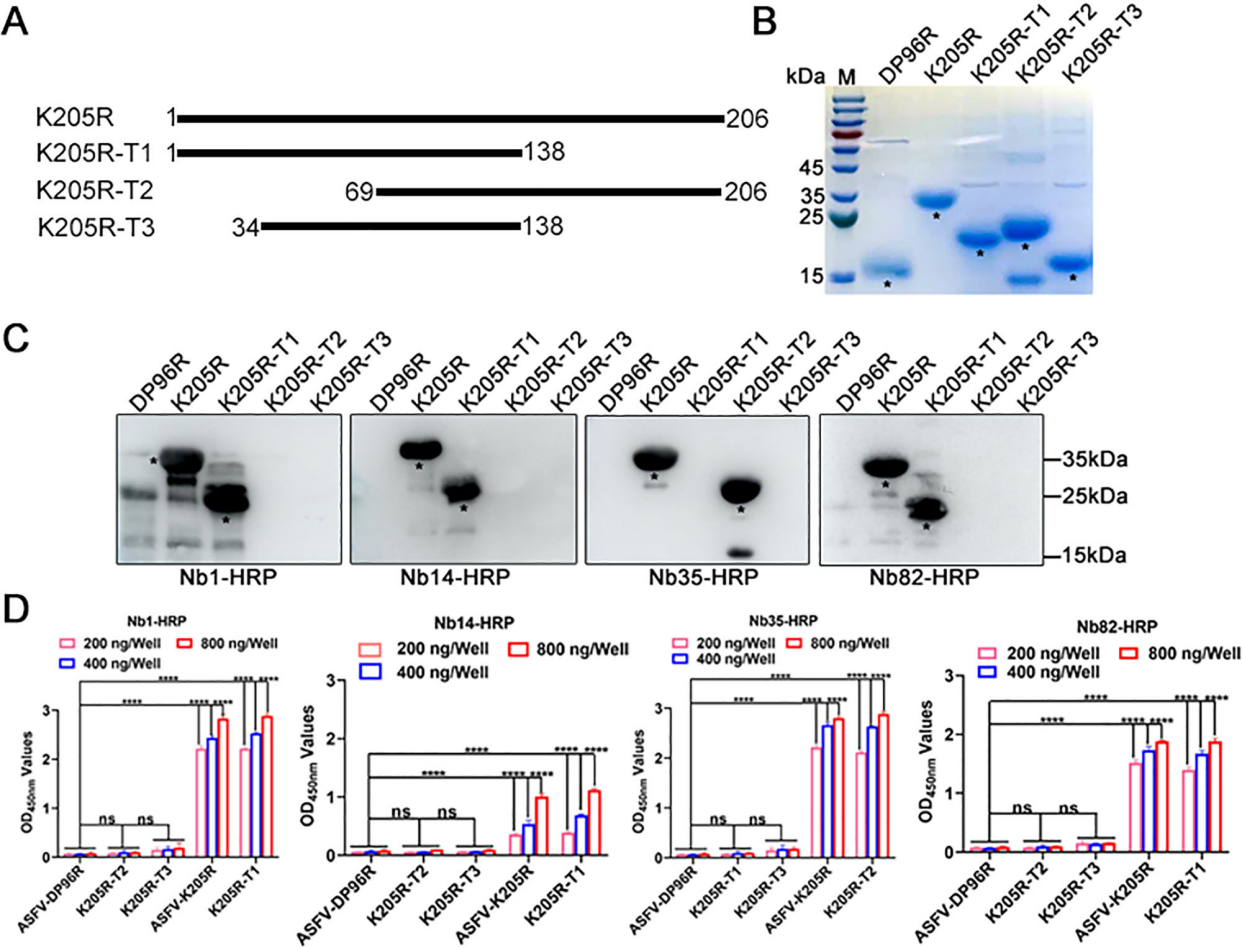
Location of the linear B-cell epitopes regions of the ASFV-K205R protein. (**A**) Schematic diagram of different truncated ASFV-K205R protein peptides. (**B**) Expression and purification of different truncated ASFV-K205R protein peptides using a prokaryotic system. (**C**) The reaction of Nb1, Nb14, Nb35, and Nb82 with the different truncated versions of ASFV-K205R protein was determined using western blotting. (**D**) Analysis of the reaction between Nb1, Nb14, Nb35, and Nb82 and different truncated versions of the ASFV-K205R protein using direct ELISA. The full-length ASFV K205R protein served as a positive control, while ASFV DP96R served as a negative control. Data are mean ± SD values of three independent results. ^****^P < 0.0001; ns, no significant. ASFV, African swine fever virus; Nb, nanobody.

To identify the epitope recognized by Nb1, Nb14, Nb35, and Nb82, different truncated ASFV-K205R proteins were designed and expressed using a prokaryotic system (Fig. 4A). SDS-PAGE analysis confirmed successful expression of these fragments with their expected sizes, which were then purified using Ni-resins (Fig. 4B). Utilizing these fragments as antigens, western blotting indicated that K205R-Nb1, -Nb14, and -Nb82 reacted with fragments spanning amino acids 1–138, excluding 69–206, and 34–138, thereby suggesting that the epitope was situated within amino acids 1–34 (Fig. 4C). Conversely, K205R-Nb35 showed reactivity with fragments amino acids 69–206, omitting 1–138 and 34–138, implying that the epitope fell within amino acids 139–206 (Fig. 4C). Direct ELISA corroborated the western blot findings (Fig. 4D). In conclusion, the results suggested that K205R-Nb1, -Nb14, and -Nb82 bound to the K205R 1–34 amino acid region, while Nb35 binds to the 139–206 amino acid region.

### Core binding sites of ASFV-K205R-Nb1, Nb14 and Nb82

**Fig 5:**
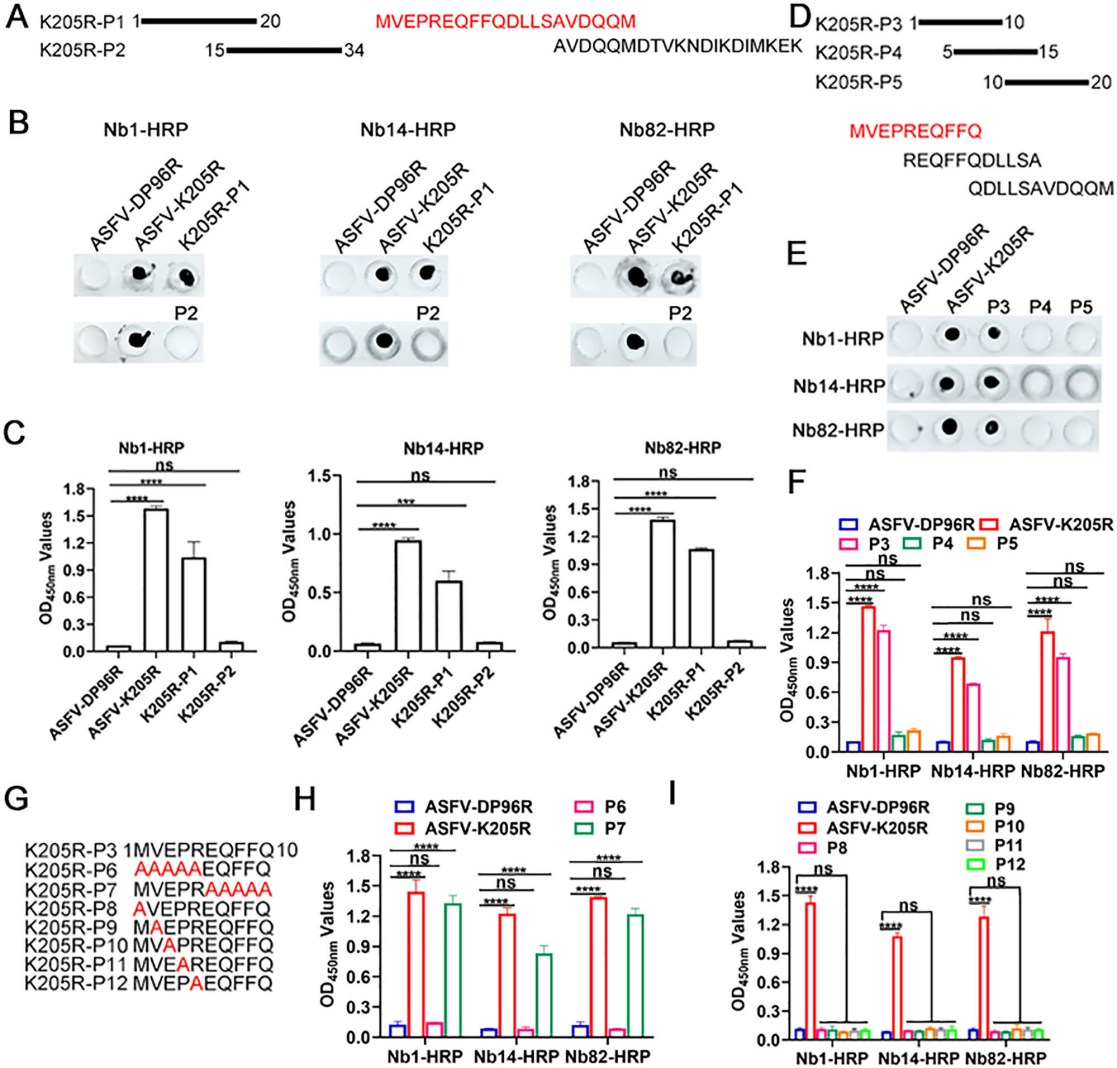
Fine mapping of the epitopes in ASFV K205R protein recognized by K205R-Nb1, -Nb14, and -Nb82. (**A, D, and G**) Schematic diagram of 11 short peptides, namely P1-P12. Reaction of K205R-Nb1, -Nb14, and -Nb82 with seven short peptides (**P1-P7**), as analyzed by (**B and E**) dot blot and (**C, F, and H**) peptide-based ELISA. The full-length ASFV K205R protein served as a positive control, while ASFV DP96R served as a negative control. (**I**) Reaction of K205R-Nb1, -Nb14, and -Nb82 with P8, P9, P10, P11, and P12 peptides by peptide-based ELISA. Data are mean ± SD values of three independent results. ^***^P < 0.001;^****^P < 0.0001; ns, no significant. ASFV, African swine fever virus; Nb, nanobody.

To further elucidate the core binding sites of Nb1, Nb14, and Nb82 to the identified linear B-cell epitopes, a series of truncated peptides from the K205R 1–34 amino acid region were synthesized and used for dot blot and peptide-based ELISA verification. First, two peptides (1–20 and 15–34 amino acids, named P1 and P2, respectively) were synthesized and employed as coated antigens to interact with Nb1-, Nb14-, and Nb82-HRP fusions (Fig. 5A). As shown in Fig. 5B, K205R-Nb1, -Nb14, and -Nb82 exhibited reactivity toward P1 1–20 amino acids but not toward P2 15–34 amino acids. Furthermore, peptide-based ELISA results also indicated that Nb1, Nb14, and Nb82 exclusively bound to K205R-P1, indicating that the epitope was situated within amino acids 1–15 (Fig. 5C).

Next, three additional peptides (1–10, 5–15, and 10–20 amino acids, named P3, P4, and P5, respectively) were synthesized and examined for their interaction with Nb1-, Nb14-, and Nb82-HRP fusions using dot blot and peptide-based ELISA (Fig. 5D). The findings revealed that Nb1, Nb14, and Nb82 interacted with P3 1–10 amino acids but not with P4 5–15 or P5 10–20 amino acids, suggesting that the epitope was confined within amino acids 1–5 (Fig. 5E and F). To ascertain the core B-cell epitopes recognized by Nb1, Nb14, and Nb82, all amino acids in the P3 1–5 or 6–10 amino acids were mutated to alanine (P6 and P7; Fig. 5G). Subsequent peptide-based ELISA results revealed that Nb1, Nb14, and Nb82 did not react with P6 but did react with P7 (Fig. 5H). Then a series of peptides with a single amino acid mutation of P3 were synthesized (P8, P9, P10, P11, and P12), and peptide-based ELISA was performed to test their reaction activity with these Nbs. The results showed that Nb1, Nb14, and Nb82 all did not react with P8, P9, P10, P11, and P12, except K205R protein (Fig. 5I). These results indicate that Nb1, Nb14, and Nb82 targeted the same B-cell epitope of ^1^MVEPR^5^.

### Core binding sites of ASFV-K205R-Nb35

**Fig 6:**
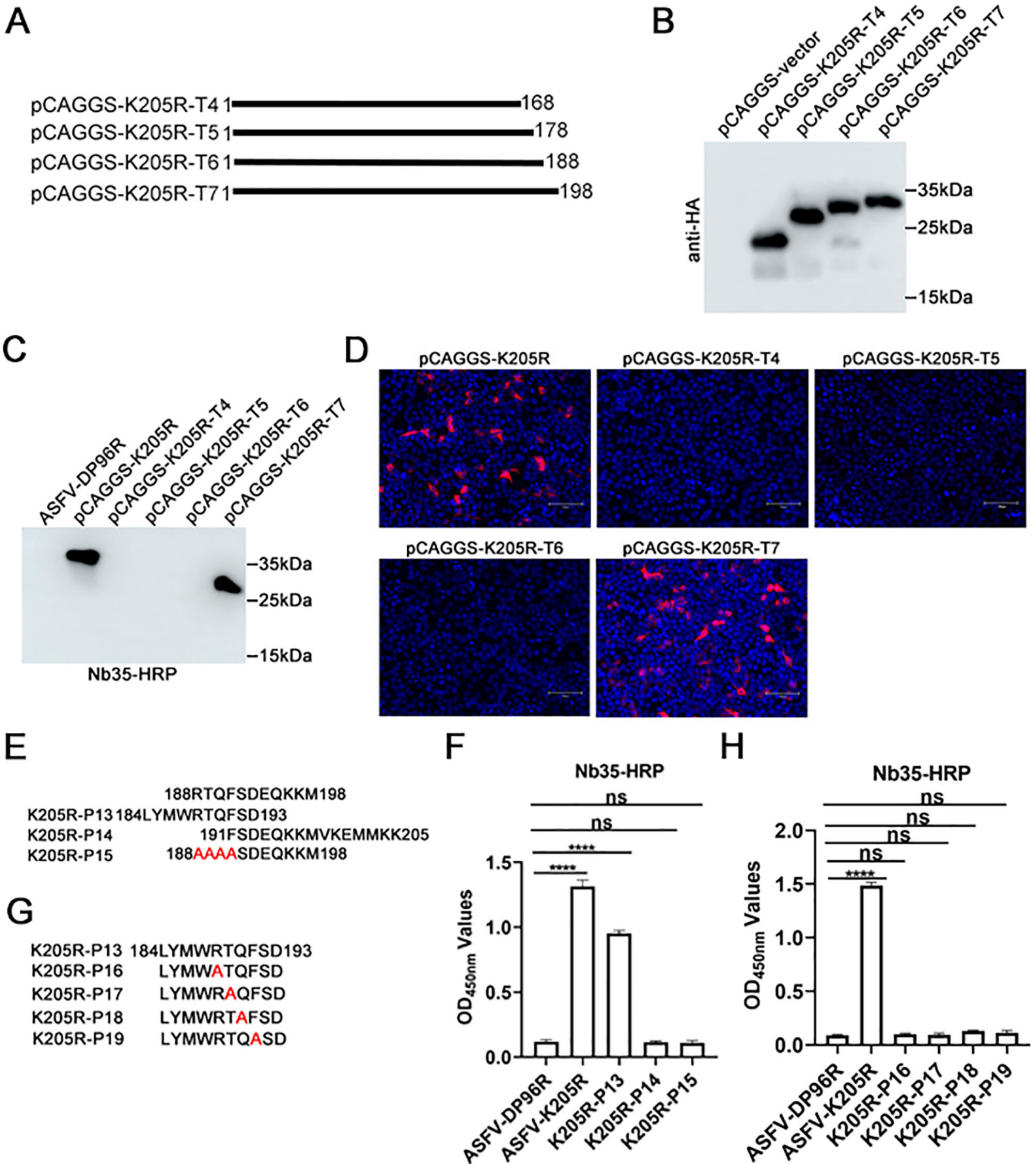
Fine mapping of the epitopes in the ASFV K205R protein recognized by K205R-Nb35. (**A**) Schematic diagram of different truncated ASFV K205R protein peptides produced in a eukaryotic expression system. (**B**) Western blot analysis of the eukaryotically expressed ASFV-K205R protein with anti-HA monoclonal antibody for detection. K205R-Nb35 reacted with different eukaryotically expressed truncated K205R protein peptides, as determined by using (**C**) western blotting and (**D**) immunofluorescence assay. (**E**) Schematic diagram of the synthesis of P13, P14, P15 truncated and mutant K205R peptides. (**F**) K205R-Nb35 reacted with different truncated and mutant K205R peptides, as revealed by peptide-based ELISA. (**G**) Schematic diagram of the synthesis of different single amino acid mutant K205R peptides. (**H**) Peptide-based ELISA detection of K205R-Nb35 reacted with P16, P17, P18, and P19. Data are mean ± SD values of three independent results. ^****^P < 0.0001; ns, no significant. African swine fever virus; Nb, nanobody.

Different truncated K205R proteins were designed and expressed in a eukaryotic system to precisely determine the epitope recognized by K205R-Nb35. According to the preliminary identification sites 139–206 amino acids of Nb35, K205R was further truncated into four segments called T4 (1–168), T5 (1–178), T6 (1–188), and T7 (1–198), and cloned into the pCAGGS-HA vector before being transfected into HEK293T cells (Fig. 6A). As shown in Fig. 6B, K205R-T4, -T5, -T6, and -T7 were successfully expressed with their expected size, as determined using anti-HA mAb as a primary antibody by western blotting. Subsequently, the reaction between Nb35 and K205R-T4, -T5, -T6, and -T7 was examined by western blot and IFA analyses. The findings indicated that K205R-Nb35 specifically interacted with T7 (1–198 amino acids), but not with T4 (1–168), T5 (1–178), or T6 (1–188 amino acids), suggesting that the epitope was localized within amino acids 188–198 (Fig. 6C and D).

Further experiments were conducted to identify the key sites for Nb35 recognition by synthesizing P13 (184–193), P14 (191–205), or P15 (RTQF mutated to AAAA) peptides as coated antigens to react with Nb35-HRP fusions (Fig. 6E). Peptide-based ELISA revealed that K205R-Nb35 specifically interacted with P13 but not with P14, besides, Nb35 also did not react with P15, confirming that the most core B-cell epitope recognized by Nb35 is ^188^RTQF^191^ (Fig. 6F). To further corroborate that ^188^RTQF^191^ was the core sequence of the identified epitope, a series of K205R peptides with single amino acid mutations were synthesized (P16, P17, P18, and P19, Fig. 6G), and then subjected to peptide-based ELISA. As shown in Fig. 6H, as a positive control, the K205R protein reacted strongly with Nb35; however, P16, P17, P18, and P19 did not react with Nb35. These results suggest that ^188^RTQF^191^ is the core sequence of the identified epitope.

### Reactivity of identified linear B-cell epitopes with inactivated ASFV-positive serum

**Fig 7:**
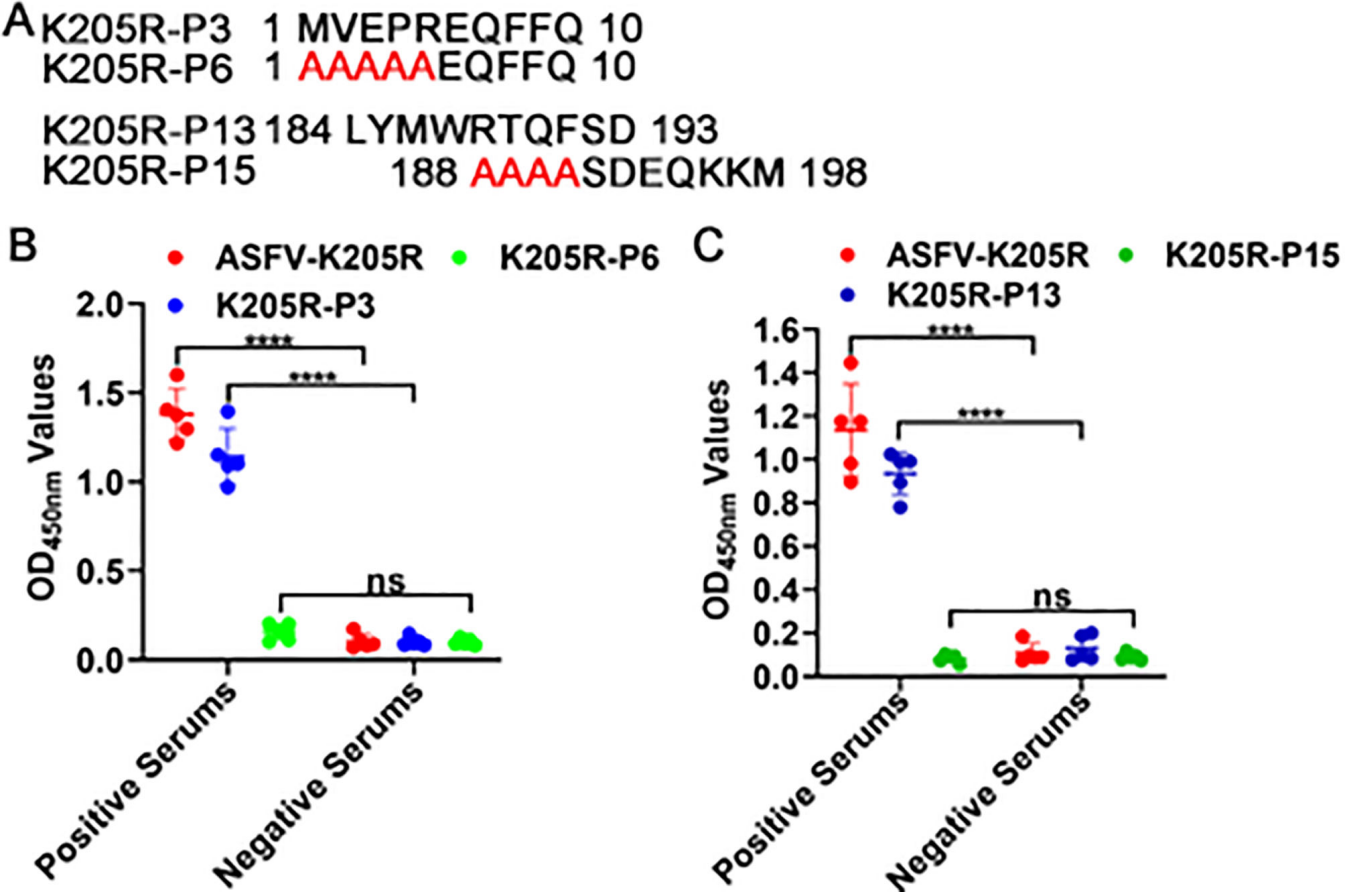
Reactivity of identified linear B-cell epitopes with inactivated ASFV-positive serum. (**A**) Schematic diagram of the synthesis of different truncated and mutant K205R peptides. (**B**) Reactivity of identified epitope ^1^MVEPR^5^ with inactivated ASFV-positive serum using peptide-based ELISA. (**C**) Reactivity of identified epitope ^188^RTQF^191^ with inactivated ASFV-positive serum using peptide-based ELISA. Five inactivated positive pig sera for anti-ASFV antibodies and five ASFV antibody-negative serums were used to test. Data are mean ± SD values of three independent results. ^***^P < 0.001;^****^P < 0.0001; ns, no significant.

To further analyze whether the identified epitopes ^1^MVEPR^5^ and ^188^RTQF^191^ are dominant epitopes, the two peptides were used as coated antigens, and whether inactivated ASFV-positive serum could recognize the two peptides was detected by peptide-based ELISA. As expected, there was a strong reactivity between the identified epitopes P3 ^1^MVEPR^5^ and P8 ^188^RTQF^191^ and all five inactivated ASFV-positive pig sera (Fig. 7A through C), while no reactivity was observed with ASFV-negative pig sera (Fig. 7A through C). These results suggest that the two identified epitopes are likely natural linear B-cell epitopes capable of eliciting a widespread humoral immune response.

### Conservation analysis of the NTD antigenic regions among ASFV epidemic isolates

**Fig 8:**
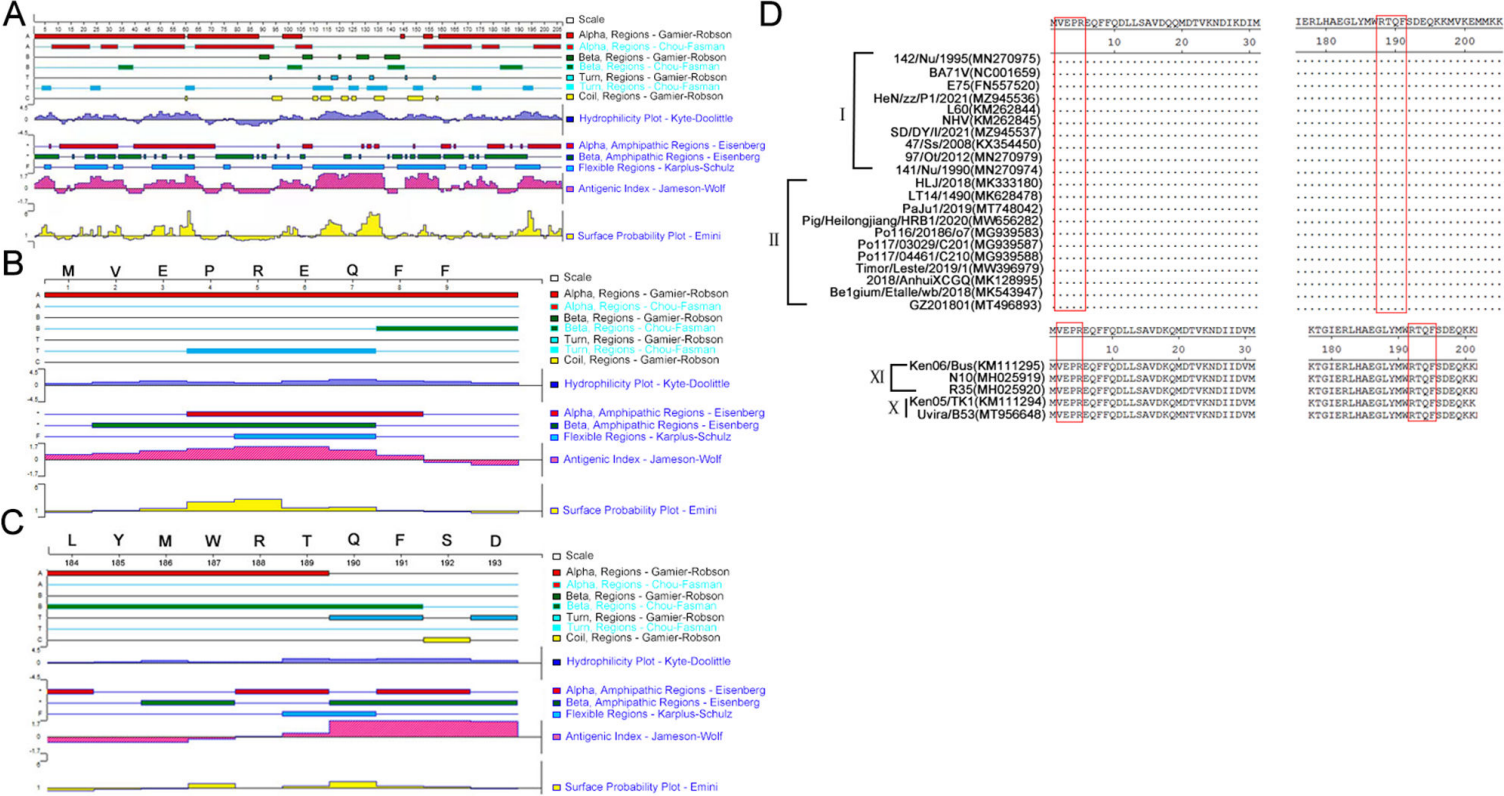
Conservation analysis of the two epitopes ^1^MVEPR^5^ and ^188^RTQF^191^. (**A**) Secondary structure analysis of ASFV K205R protein using DNASTAR Protean software. Secondary structure analysis of epitopes ^1^MVEPR^5^ (**B**) and ^188^RTQF^191^ (**C**) using DNASTAR Protean software. (**D**) Conservation analysis of the ^1^MVEPR^5^ and ^188^RTQF^191^ antigenic regions among ASFV epidemic isolates. The red box indicated the antigenic region included two epitopes ^1^MVEPR^5^ and ^188^RTQF^191^.

The epitopes ^1^MVEPR^5^ and ^188^RTQF^191^ were characterized using DNASTAR Protean software. As shown in Fig. 8A, the distribution region of the K205R protein secondary structure (including α-helix, β-fold, β-corner, and random coil), hydrophilicity, antigenic index, and surface probability were predicted using DNASTAR software. The epitope ^1^MVEPR5 was found at the N-terminal region of the K205R protein, exhibiting a high antigenic index and being distributed on the surface (Fig. 8B). Similarly, epitope ^188^RTQF^191^, located at the C-terminal region of the K205R protein, displayed an antigenic index, and was also distributed on the surface (Fig. 8C).

Furthermore, to analyze the amino acid conservation of the epitopes ^1^MVEPR^5^ and ^188^RTQF^191^, the amino acid 2–5 and 188–191 regions of K205R proteins from different ASFV strains were aligned. The sequence alignments revealed that the two epitopes ^1^MVEPR^5^ and ^188^RTQF^191^ were conserved among various genotype I, II, IX, and X ASFV strains (Fig. 8D).

### Compound docking of K205R peptide and Nb

**Fig 9:**
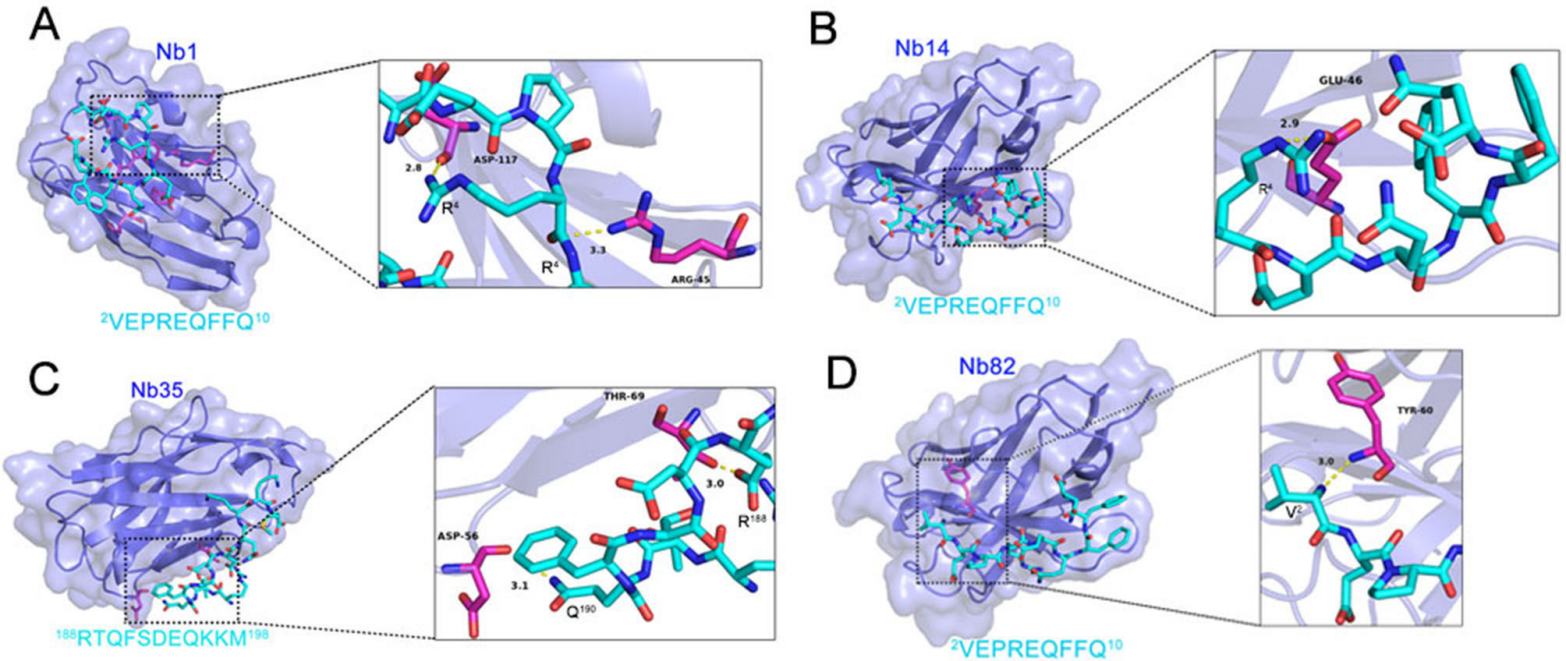
Predict complex structure using molecular docking. (**A**) Molecular docking of complex Nb1 and the identified epitope ^1^MVEPREQFFQ^10^. (**B**) Molecular docking of complex Nb14 and the identified epitope ^1^MVEPREQFFQ^10^. (**C**) Molecular docking of complex Nb35 and the identified epitope ^188^RTQFSDEQKKM^198^. (**D**) Molecular docking of complex Nb82 and the identified epitope ^1^MVEPREQFFQ^10^. K205R epitope is shown as a cyan stick, the nanobody is represented as a slate cartoon model, and their binding sites are shown as magentas stick structures. The hydrogen bond is depicted by a yellow dashed line.

To preliminarily analyze the complex structure of the identified epitopes of K205R and Nb, molecular docking was performed. The interactions between Nbs and epitopes were analyzed, and all functional residues were identified and classified according to their interactions. There were multiple groups of residues used to form interactions between receptor protein and ligand, such as the hydrogen bond formed by ARG45 and ASP117 of Nb1 and ligand 4R; the hydrogen bond formed by GLU46 of Nb14 and ligand 4R; the hydrogen bond formed by THR69 of Nb35 and ligand 188R, and ASP56 of Nb35 and ligand 190Q; and the hydrogen bond formed by TYR60 of Nb82 and ligand 2V. With these interaction forces, the binding energy of the protein-ligand complex was calculated to be −4.6 kcal/mol for Nb1 and ^1^MVEPREQFFQ^10^ (Fig. 9A), −5.8 kcal/mol for Nb14 and ^1^MVEPREQFFQ^10^ (Fig. 9B), −4.2 kcal/mol for Nb35 and ^188^RTQFSDEQKKM^198^ (Fig. 9C), and −5.9 kcal/mol for Nb82 and ^1^MVEPREQFFQ^10^ (Fig. 9D), which all show a good performance.

## DISCUSSION

ASF is characterized by high contagiousness, high infection rate, and rapid onset, and an outbreak of ASF can cause a large number of pigs to be infected and die, which brings a devastating blow to the global pig industry (27, 28). Currently, there is a lack of adequate and effective prevention and control measures against ASF, and infections often require the culling of large numbers of pigs. Vaccines remain the most effective strategy for preventing and controlling infectious diseases. B-cell epitopes, which are surface-accessible clusters of amino acids recognized by specific antibodies, play a crucial role in inducing a cellular or humoral immune response (29). The identification of novel epitopes is essential for developing diagnostic tools, effective vaccines, and therapeutic antibodies (30). This approach has been successfully applied to the development of vaccine candidates against different viruses (31, 32). P30, p54, and p72 can induce high levels of antibodies and are commonly used for serological detection of ASFV. These detection methods can detect serum antibodies as early as 7 days after ASFV infection (13). K205R protein is one of the main antigens of ASFV, which can induce a strong immune response in the body. Research has found that K205R is present in the early stages of ASFV infection and begins to be expressed 4 hours after viral infection (11). Therefore, the K205R protein is of great significance for the early diagnosis of ASFV. K205R can also induce antibody production in the early stages of ASFV infection, even earlier than p30, p54, and p72, and has significant advantages in antibody detection.

ASFV is complex and contains multiple proteins, presenting challenges in vaccine development. Therefore, exploring the function of ASFV proteins will help in the development of vaccines. At present, most of the protein function is still unknown, and a few of the existing protein studies reported in the literature involve the function of K205R. K205R, a non-structural protein of ASFV, is abundantly expressed during the early stages of viral infection and induces a strong immune response (10, 11). Identifying novel epitopes within K205R is crucial for the advancement of ASFV subunit vaccines and offers insights into the function of the K205R protein and the pathogenesis of ASFV. Our previous study demonstrated that Nb1, Nb35, and Nb82 could block the reaction of inactivated ASFV-positive serum with K205R protein, suggesting the recognition of natural dominant epitopes (21). These epitopes may play an important role in vaccine development.

Based on the particular structure of Nbs, which are good at recognizing hidden epitopes of antigens compared with traditional antibodies (33). Four pre-screened Nbs against the K205R protein were used as tools in the present study to identify the novel dominant epitopes of ASFV K205R protein. The findings demonstrated that all four Nbs recognized the linear epitopes of K205R (Fig. 2B). Although all four Nbs did not react with another ASFV protein DP96R, some unspecific bands indeed exist in the DP96R lane (Fig. 2B). We speculated that after purification by nickel column, there were still some residual unspecific proteins, and these unspecific proteins could undergo non-specific reactions with these Nbs. Considering that the protein modification system in eukaryotes is much more complex than that of prokaryotes, whether the four Nbs could also recognize eukaryotically expressed K205R protein was explored. The western blot and IFA results indicated that the four Nbs exhibited reactivity toward the K205R protein in a eukaryotic expression system (Fig. 2F and G), suggesting that the difference in modification between eukaryotic and prokaryotic expression does not affect the recognition of antigen epitopes by Nb1, Nb14, Nb35, or Nb82. Ultimately, these findings further confirmed that all four Nbs were capable of recognizing linear epitopes, regardless of the expression system used (Fig. 2).

In the present study, two epitopes, ^1^MVEPR^5^ and ^188^RTQF^191^, were identified, of which epitope ^188^RTQF^191^ was reported for the first time, while epitope ^1^MVEPR^5^ had been reported before, but the current findings further enhanced its accuracy (34). Notably, three Nbs (Nb1, Nb14, and Nb82) were found to recognize the same epitope, ^1^MVEPR^5^, while Nb1 competitively bound to K205R protein with ASFV-positive serum (21), suggesting that the ^1^MVEPR^5^ epitope was likely to be the dominant epitope. It has been previously reported that ASFV can be divided into eight serogroups according to the CD2v gene and 24 genotypes based on the C-terminal variable region sequence B646L gene of the capsid protein p72 (35). Both genotypes I and II ASFVs have been found in pigs in China, and more importantly, highly lethal recombinant ASFVs of genotypes I and II have been also detected in pigs recently, which has increased the difficulty of AFS prevention and control (36). The two epitopes ^1^MVEPR^5^ and ^188^RTQF^191^ were identified to be conserved among various ASFV strains of genotypes I, II, IX, and X (Fig. 7). In addition, the two epitopes ^1^MVEPR^5^ and ^188^RTQF^191^ could react with inactivated genotype II ASFV-positive sera, indicating that the two epitopes were natural linear B-cell epitopes (Fig. 7). Given that the two epitopes recognized by the four Nbs-HRP are highly conserved among different ASFV strains, we speculate that these Nbs-HRP can also react with target cells infected by other ASFV strains, similar to the results in Fig. 3A.

In conclusion, the two epitopes ^1^MVEPR^5^ and ^188^RTQF^191^ of K205R protein were identified by Nbs as a tool in the current study, with a higher degree of conservation across various ASFV strains. Notably, the epitope ^188^RTQF^191^ was reported for the first time in the present study. The two epitopes could react with inactivated ASFV-positive sera, indicating that the two epitopes were natural linear B-cell epitopes. Specific Nbs and Nb-based diagnostic assays would be crucial tools to aid in the control and prevention of ASF outbreaks.
